# Opioid Use Disorder Education for Students and the Future of Opioid Overdose Treatment

**DOI:** 10.2196/37081

**Published:** 2022-07-18

**Authors:** Neha Balapal, Amala Ankem, Saishravan Shyamsundar, Shuhan He

**Affiliations:** 1 City University of New York School of Medicine New York, NY United States; 2 Lab of Computer Science Massachusetts General Hospital Boston, MA United States; 3 Geisinger Commonwealth School of Medicine Scranton, PA United States

**Keywords:** opioid use disorder, students, buprenorphine, education, public health, opioid, health care providers, healthcare providers, medication-assisted treatment, youth, substance use, opioid agonist, overdose

## Abstract

Opioid use disorder (OUD) is a major public health concern in the United States. The opioid crisis has taken hundreds of thousands of lives in the past 20 years, and it is predicted to take millions more. With the rising death tolls, it is essential that health care providers are able to use proper tools to treat OUD efficiently and effectively through medication-assisted treatment (MAT), particularly buprenorphine. Despite changes to buprenorphine regulations making it more accessible, clinicians have been slow to use buprenorphine to treat OUD. We believe that training student clinicians in evidence-based MAT and buprenorphine practices will address the training and competence barriers that hinder clinicians from prescribing buprenorphine to treat OUD. Students are in an ideal position to receive and benefit from this training and influence the medical community to better treat OUD.

The opioid epidemic and substance use disorders have long been a major public health crisis in the United States. From 1999 to 2019, there have been approximately 500,000 opioid overdose deaths in the United States [1]. The opioid epidemic has had various social and economic effects on US society, all of which have been recently exacerbated by the COVID-19 pandemic’s influence on the lack of access to treatment and mental health challenges of those with opioid use disorder (OUD). Predicted overdose deaths for the upcoming years offer a grim outlook [2] despite the steady decline of opioid prescribing since 2012 and the all-time low reached in 2020 [3]. The United States may see more than 1.2 million OUD overdose deaths in the upcoming decade if urgent action is not taken [4].

Buprenorphine is a US Food and Drug Administration (FDA)–approved medication used as a medication-assisted treatment (MAT) by acting as a partial opioid agonist. It is used to treat OUD along with a comprehensive treatment program that includes various behavioral therapies and counseling. Buprenorphine is a key step toward increasing treatment access for patients with OUD [5]. However, the ability of a clinician to prescribe buprenorphine is contingent upon completion of specialized training and obtaining their “X” designation from the Drug Enforcement Administration (DEA-X). The X waiver was necessary in part because training for opioid overdose treatment and prevention using evidence-based MAT practices allows clinicians to be more prepared to treat patients with OUD. Due to the many accessibility barriers to obtaining an X waiver in the past, only about 7% of physicians in the United States have done so, which limits the ability to care for patients with OUD [6]. Ability to care is further limited in rural areas where there are even fewer buprenorphine-waivered clinicians.

Get Waivered (GW) is a project started in 2017 that aims to address the opioid crisis by encouraging and facilitating more clinicians to get their X waiver. GW’s work focuses on behavioral nudges to address the barriers identified as to why clinicians do not obtain their X waiver: (1) absence of a social norm, (2) hassle bias in obtaining the waiver, and (3) a lack of salience in treating OUD [7]. With the COVID-19 pandemic, social distancing practices have limited accessibility to traditional medical education, including the X waiver training courses. Thus, there has been a movement toward online platforms for medical education. To adapt to these circumstances, GW started delivering and hosting free, nationwide, digitally delivered, and interactive X waiver educational training courses. Therefore, clinicians are able to obtain their X waiver using a live, synchronized, and interactive digital platform while learning evidence-based best practices for buprenorphine prescription.

Despite recent policy changes regarding the X waiver, buprenorphine prescription education is still important for clinicians, especially student clinicians (those enrolled in health care training programs). While the legal barriers may be overcome, clinicians still need the opportunity and motivation to prescribe buprenorphine [8]. To have effective change, clinicians must be properly educated on how to use their waiver and be able to look for prescription opportunities. This training needs to start in undergraduate or medical education settings so that it can create a cultural shift [8]. It has been shown that educational interventions in opioid overdose prevention have led to students being more confident and prepared to act [9], and this can be applied to buprenorphine training as well. There is currently no unified approach to teaching student clinicians about buprenorphine administration, but training would provide meaningful education while also increasing the pool of future buprenorphine-prescribing clinicians [10]. Although medical students cannot use their waiver education until entering their residency programs, they still interact with patients with OUD during clerkship and various clinical experiences; their OUD and buprenorphine knowledge can still be vital for encouraging patients to seek treatment, decreasing stigma surrounding OUD, and encouraging superiors to take action [10]. Thus, X waiver education is still a vital component for combating the opioid epidemic.

In the past few years, there have been several changes made in the practical guidelines for obtaining an X waiver. The initial guidelines in place prior to regulatory changes dictated that a physician could give buprenorphine to a patient experiencing opioid withdrawal in a hospital setting, but prescriptions for buprenorphine in an outpatient or clinic setting require an X waiver. The process to obtain an X waiver required the completion of an 8-hour education training course for physicians and a 24-hour education training course for advanced practice clinicians. In 2016, policy changes led to an expansion of the patient limit from 100 patients after the first-year postwaiver completion to 275 patients [11]. These policy changes also expanded X waiver access to include nurse practitioners and physician assistants; the clinician list of people who can now obtain an X waiver include physicians, nurse practitioners, physician assistants, clinical nurse specialists, certified registered nurse anesthetists, and certified nurse midwives. On January 14, 2021, the US Department of Health and Human Services under the Trump administration announced the elimination of the X waiver requirement altogether for buprenorphine prescription for physicians only [12]. This decision was then canceled under the Biden administration due to legal challenges that may be faced, and the policy was reverted and eventually changed in April 2021 [13]. As of April 28, 2021, practice guidelines dictate that clinicians can apply for exemption and do not need to undergo the educational training requirement of an X waiver. This process involves submitting a state-issued license, valid registration, and a notice of intent to the Substance Abuse and Mental Health Services Administration [5]. The alternative notice-of-intent process only applies to clinicians who are treating ≤30 patients; providers who wish to treat >30 patients still need to undergo the education training course.

While the policy change in April 2021 has allowed for an unprecedented expansion of the buprenorphine waiver program, the results of this increased access are still forthcoming. A recent study [11] found that while the number of clinicians who can prescribe buprenorphine and buprenorphine prescribing increased in California between 2010 and 2018, it has not necessarily been associated with changes in opioid-related health outcomes. There are still many barriers to adequate OUD treatment including lack of training and support among clinicians, care coordination, cost of treatment, and stigma [11]. Furthermore, even though buprenorphine-prescribing clinicians have increased since 2016, many choose not to be publicly listed, which limits access to treatment since many clinicians may not take new patients [14]. New clinicians have been shown to be slow to use their new buprenorphine prescription abilities, often due to a lack of confidence, fear of the prescription being misused, lack of understanding of addiction, and lack of peer pressure. This has led to very few short-term changes from buprenorphine access expansion [15].

It is imperative that student clinicians (especially medical students and residents) obtain X waivers as part of their educational training. Basic OUD prevention and treatment competence among those licensed to prescribe buprenorphine may lead to a large increase in buprenorphine prescribing and improvement of opioid-related health outcomes. These ideas have been recommended by numerous studies that have indicated that lack of training is one of the foremost barriers to buprenorphine prescription [7]. Many competence and training barriers can be addressed through brief short education and networking opportunities, which is what GW offers. In fact, in 2018, the Substance Abuse and Mental Health Services Administration started to provide funding for medical schools willing to adjust their curriculum to fulfill waiver training requirements, but only one school has done so [10]. Some recent pilot studies in the United States have shown that integrating waiver training for medical students does increase knowledge, interest, and confidence in diagnosing and treating OUD [16,17], and calls attention to the need for a standardized, nationwide course, as there is currently none. A key advantage of GW courses is that even though the requirements for buprenorphine prescription have been relaxed, GW can provide mentorship and education in a brief, free, virtual, and interactive setting that boosts confidence and arms clinicians with the tools they need to effectively use their X waiver. Clinicians and student clinicians have the opportunity to interact with professional peers from around the country, which facilitates networking and discussion of shared values and interests. Mentorship and education have been mentioned as the key resources to increase buprenorphine prescribing by clinicians themselves [18]. Furthermore, the availability of remote education has opened the unique possibility of bridging the disparity in provider availability between urban and rural areas.

Student clinicians are in an ideal position to receive buprenorphine prescription training since many of them are already learning about OUD as part of their psychiatric training and can easily benefit from education on clinical applications. Additionally, students can be more easily empowered to bring change into the field of medicine since they are already involved in various advocacy and research campaigns. Student clinicians can be molded to bring a new mindset about OUD treatment into their clinical rotations and their future workplaces, and this has already been seen with the rise of social media platforms discussing medical topics and the popularity of “medical influencers.” Implementation of GW courses or similar workshops in the curriculum of medical school, nursing school, and residency training programs have the ability to influence a generational change in the perceptions and feasibility of OUD treatment without requiring significant time or money, a vast difference from previous in-person courses. Now that regulatory policies have been relaxed, it is high time to address the other barriers to evidence-based OUD treatment and make meaningful changes. The future of mitigating the opioid crisis lies in using innovative, broad-reaching networks like social media and behavioral nudges to enhance education and connection among clinicians, and GW is in an ideal position to bring about that change.

## Abbreviations

DEA-X or X waiver: “X” designation from the Drug Enforcement Administration
FDA: US Food and Drug Administration
FORE: Foundation for Opioid Response Efforts
GW: Get Waivered
MAT: medication-assisted treatment
OUD: opioid use disorder
